# Recombinant human adenovirus type 5 synergizes with anti-PD-L1 antibody to promote anti-hepatocellular carcinoma effects through multilevel remodeling of the immune microenvironment

**DOI:** 10.3389/fimmu.2026.1746243

**Published:** 2026-03-24

**Authors:** Qiu Zhao, Min Xiao, Jian Ma, Xiaoqian Li, Cong Fu, Wenjing Ji, Lingna Ni, Jialin Fan, Qianqian Gao, Yanzhi Bi

**Affiliations:** 1 Department of Oncology, Changzhou City Fourth People’s Hospital, Changzhou, China; 2 Department of Pathology, Changzhou City Fourth People’s Hospital, Changzhou, China

**Keywords:** CD274, CD4+ T cell, CD8+ T cell, hepatocellular carcinoma, IFN-γ, M1 macrophages, recombinant human adenovirus type 5 (H101), STAT1

## Abstract

**Background:**

Overcoming resistance to immune checkpoint inhibitors (ICIs) remains a critical challenge in the treatment for hepatocellular carcinoma (HCC).

**Methods:**

Our study evaluated the combined efficacy of the recombinant human adenovirus type 5 (H101) and an anti-PD-L1 antibody (APA) in C57BL/6N mouse model. Gene expression of STAT1 and CD274 (PD-L1) was analyzed by qPCR, and protein levels of PD-L1 and p-STAT1 were assessed via western blot and immunohistochemistry. IFN-γ secretion and immune cell infiltration were measured by ELISA and flow cytometry, respectively.

**Results:**

*In vitro*, H101 inhibited the proliferation and invasion and IFN-γ induced apoptosis in HCC cells, with IFN-γ upregulating the expression of STAT1 and CD274. Transfection with si-STAT1 significantly reduced the levels of both STAT1 and CD274/PD-L1. *In vivo*, the combination of H101 and APA demonstrated stronger anti-tumor activity, with upregulated IFN-γ expression and increased infiltration level of CD8+ T cell. Notably, si-STAT1 transfection reduced the expression of p-STAT1 and PD-L1, thereby diminishing the efficacy of both APA monotherapy and the H101+APA combination treatment. The combination therapy significantly reshaped the tumor microenvironment (TME) after si-STAT1 transfection.

**Conclusion:**

The combination of H101 and APA exhibited excellent anti-HCC efficacy, which may reshape the TME partially through the IFN-γ/STAT1/PD-L1 axis. These findings provide a promising strategy to overcome resistance to ICIs in HCC treatment.

## 1 Introduction

Primary liver cancer is one of the most common malignant tumors, with hepatocellular carcinoma (HCC) accounting for 75%-85% of all primary liver cancer cases (1). Immune checkpoint inhibitors (ICIs) have demonstrated remarkable efficacy across various malignancies and are now extensively employed in the clinical management of HCC (2). Immune checkpoints, including the PD-1 and its ligands (PD-L1/PD-L2), CTLA-4, LAG3, and TIM3, serve critical physiological roles in maintaining immune homeostasis and preventing autoimmune diseases (3). For instance, PD-L1 inhibitors, such as atezolizumab and durvalumab, have gained prominent clinical application in HCC treatment regimens (4–6). However, the immune characteristics of HCC exhibit high heterogeneity, with certain subtypes manifesting as “cold tumors” characterized by T-cell deficiency, infiltration of immunosuppressive cells, and impaired antigen presentation, collectively hindering effective antitumor immune responses (7, 8). To address this challenge, remodeling the tumor microenvironment (TME) to covert the immunosuppressive “cold tumor” phenotype into a “hot tumor” state has emerged as a pivotal therapeutic strategy, which aims to enhance T-cell infiltration, restore antigen-presentation function, and counteract immunosuppressive signals, thereby improving the responsiveness of HCC to immunotherapy (9, 10).

As a novel form of anti-tumor immunotherapy, oncolytic viruses (OVs) replicate specifically in tumor cells and simultaneously target multiple elements of the TME to remodel it into an antitumor environment (11). Hence, OVs have emerged as a promising modality for combination therapy to overcome the limitations seen with ICIs (12). Recombinant human adenovirus type 5 (H101), a genetically engineered oncolytic adenovirus derived from an adenovirus type 5 (Ad5) backbone, was constructed via genetic recombination technology with deletions in the E1B-55kD gene and partial E3 region (13). Notably, H101 selectively replicates and exerts anti−tumor effects in p53−deficient tumor cells, including HCC cells, while barely affecting normal cells with intact p53 function. Through viral replication and tumor cell lysis, H101 triggers the immunogenic death of tumor cells, thereby potentiating the anti-tumor immune response (14, 15). A case report indicated that H101 overcame immune resistance in advanced esophageal squamous cell carcinoma by modulating the TME (13). A retrospective study suggested that combination of H101 with transcatheter arterial embolization sequential thermal ablation was safe and effective for HCC (16). Additionally, a clinical study revealed an acceptable safety profile for the combination of M1-c6v1 with camrelizumab and apatinib in patients with advanced HCC (17). Although OVs may sensitize “cold” tumors to ICIs by modulating the TME, there remains a paucity of substantial evidence to elucidate the underlying regulatory mechanism of H101 combined with ICIs in the treatment of HCC.

In this study, we hypothesize that OVs can not only directly kill HCC cells but also enhance the efficacy of ICIs by remodeling the immunosuppressive TME. To verify this hypothesis, we investigated the therapeutic effects of H101 (Oncorine) combination with an anti-PD-L1 antibody (APA) in HCC cell lines and mouse models, and explore the underlying regulatory mechanism of combined therapy in HCC treatment. Our study may provide a promising approach to overcoming immunosuppressive resistance in HCC and possesses considerable potential for clinical translation.

## 2 Materials and methods

### 2.1 Cell culture and oncolytic adenoviruses

The Huh-7, Hepa1–6 and H22 cells were purchased from the cell bank of the Chinese Academy of Sciences. Huh-7 and Hepa1–6 cells were cultured in 90% DMEM high-glucose medium (Procell), while H22 cells were cultured in 90% RPMI-1640 medium (Procell). The culture medium was supplemented with 10% fetal bovine serum (Cell Box, CF-01P-02-S). All cell lines were incubated in a humidified environment at 37°C with 5% CO_2_. H101 was purchased from Shanghai Sunway Biotech (Shanghai, China).

### 2.2 Cytotoxicity assay

A 100 µL aliquot of the cell suspension was seeded into a 96-well plate. Control solution and diluted H101 were then added to designated wells. After 24 or 48 h of treatment, 10 µL of MTT solution (Biosharp) was added to each well, followed by 150 µL of DMSO to dissolve the formazan crystals. The plate was gently shaken for 10 min, and absorbance was measured at 490 nm using a microplate reader.

### 2.3 Cell transfection

Huh-7 and Hepa1–6 cells were seeded in 12-well plates. When cell confluence reached 50%, cell transfection was performed. The siRNA-mate transfection reagent was brought to room temperature and gently mixed prior to use. 200 μL of DMEM was added to a sterile EP tube. 50 pmol of si-STAT1 was added and mixed thoroughly. Then, 4 μL of siRNA-mate transfection reagent was added to the tube, followed by rapid vortexing for 10 s to ensure thorough mixing. The mixture was incubated at room temperature for 10 min to allow the formation of transfection complexes between si-STAT1 and the transfection reagent. 200 μL of the transfection mixture was added to each well. The plate was gently shaken to ensure even distribution, and the cells were incubated at 37 °C. Samples were collected 24 h later for qPCR detection.

### 2.4 qPCR analysis

Cells from culture plates were harvested, and total RNA was extracted using an RNA isolation kit (CWBIO, CW3711S) according to the manufacturer’s protocol. RNA purity and concentration were determined by measuring A_260_/A_280_ ratios (acceptable range: 1.8–2.0) using a NanoDrop spectrophotometer. One-step RT-qPCR was performed with a commercial kit (CWBIO, CW0659S) to quantify mRNA expression of target genes (STAT1, CD274).

### 2.5 Western blot analysis

Cells were collected and centrifuged at 12,000 rpm for 20 min at 4 °C to obtain the supernatant containing cellular proteins. The extracted proteins were mixed with 5× loading buffer and heated at 105 °C for 5 min in a dry bath incubator. Equal amounts of proteins were separated by electrophoresis and transferred onto a PVDF membrane. The membrane was then incubated overnight at 4 °C with primary antibodies against STAT1 and PD-L1, followed by incubation with HRP-conjugated secondary antibodies. Protein bands were visualized using the iBright™ CL750 imaging system. Catalog number for molecular weight standard: Thermo Fisher Scientific PageRuler™ Plus Prestained Protein Ladder, 10 to 250 kDa, catalog number #26616.

### 2.6 Flow cytometry analysis

Tumor tissues were cut into small pieces (1–3 mm^3^) and processed into single-cell suspensions. For CD4+ and CD8+ T cell analysis, the following antibodies were used: CD45-PerCP (BioLegend, 109826), FITC-CD3 (BioLegend, 100204), APC-CD4 (BioLegend, 100412), and PE-CD8 (BioLegend, 100708). For B cells and NK cells, the antibodies included CD45-PerCP (BioLegend, 109826), FITC-CD3 (BioLegend, 100204), APC/Fire™ 810-CD19 (BioLegend, 115578), and PE-CD49b (BioLegend, 108908). For macrophage characterization, the staining panel consisted of PE-F4/80 (BioLegend, 157303), FITC-CD11b (BioLegend, 101205), APC-CD86 (BioLegend, 105011), Alexa Fluor^®^ 700-CD206 (BioLegend, 141733), fixation buffer (BioLegend, 420801), and intracellular staining permeabilization wash buffer (BioLegend, 421002).

### 2.7 HE, immunohistochemistry

The tumor tissue was fixed with 4% paraformaldehyde, then dehydrated, transparentized, and embedded to. A blocking solution was added, and incubation was carried out at room temperature for 10–20 minutes to block non-specific binding sites. Anti-p-STAT1 (Affinity, 1:600) and anti-PD-L1 (Proteintech; 1:1400) were dropped onto the slides to completely cover them. A secondary antibody was added and incubated at room temperature for 1 hour. When brownish-yellow staining appeared, the reaction was immediately stopped by rinsing with distilled water. The cell nuclei were stained with hematoxylin for 30–60 seconds, then differentiated with hydrochloric acid alcohol, sealed with neutral gum, and observed under a microscope.

### 2.8 ELISA

According to the instructions of the ELISA kit (JONLNBIO, JL10967), the subsequent operations were carried out. The tumor tissue was mixed with the extraction solution at a ratio of 1:9, and then the tissue homogenate was prepared. The homogenate was centrifuged at 5000×g for 10 min, and the supernatant was taken for detection.

### 2.9 Mice

The mice were housed under controlled environmental conditions with temperature maintained at 19-26 °C, relative humidity at 40-70%, and a 10-h light/14-h dark cycle, with free access to standard rodent chow and water. All experimental mice were 6–12 weeks old with equal gender distribution across groups. C57BL/6 mice were obtained from Vital River Labs and acclimatized in our laboratory animal facility. Humane endpoints were strictly enforced, with euthanasia by cervical dislocation performed when tumor volume exceeded 2000 mm^3^ or treatment duration reached 14 days. Experiments were immediately terminated if any tumor dimension reached 15 mm in length, width or depth, or upon observation of any signs of distress including reduced food/water intake, significant weight loss (>20%), hunched posture, or ruffled fur. All procedures were conducted in full compliance with the ARRIVE guidelines for reporting animal research.

### 2.10 Statistical analysis

Statistical software Prism 9.5 (GraphPad) was used for statistical analysis. Student’s t test was utilized to compare two independent groups, and one-way ANOVA model with the correction of Tukey was utilized to compare three or more groups. Data are shown as mean ± SD. Differences associated with p <0.05 were considered significant.

### 2.11 Ethical approval

For *in vivo* assays, ethical approval was obtained from the Jiangsu Kerbio Medical Technology Group Co, Ltd. Laboratory Animal Management Committee (Ethics Committee).

## 3 Results

### 3.1 H101 exhibits cytotoxic effects on HCC cell lines

**Figure 1:**
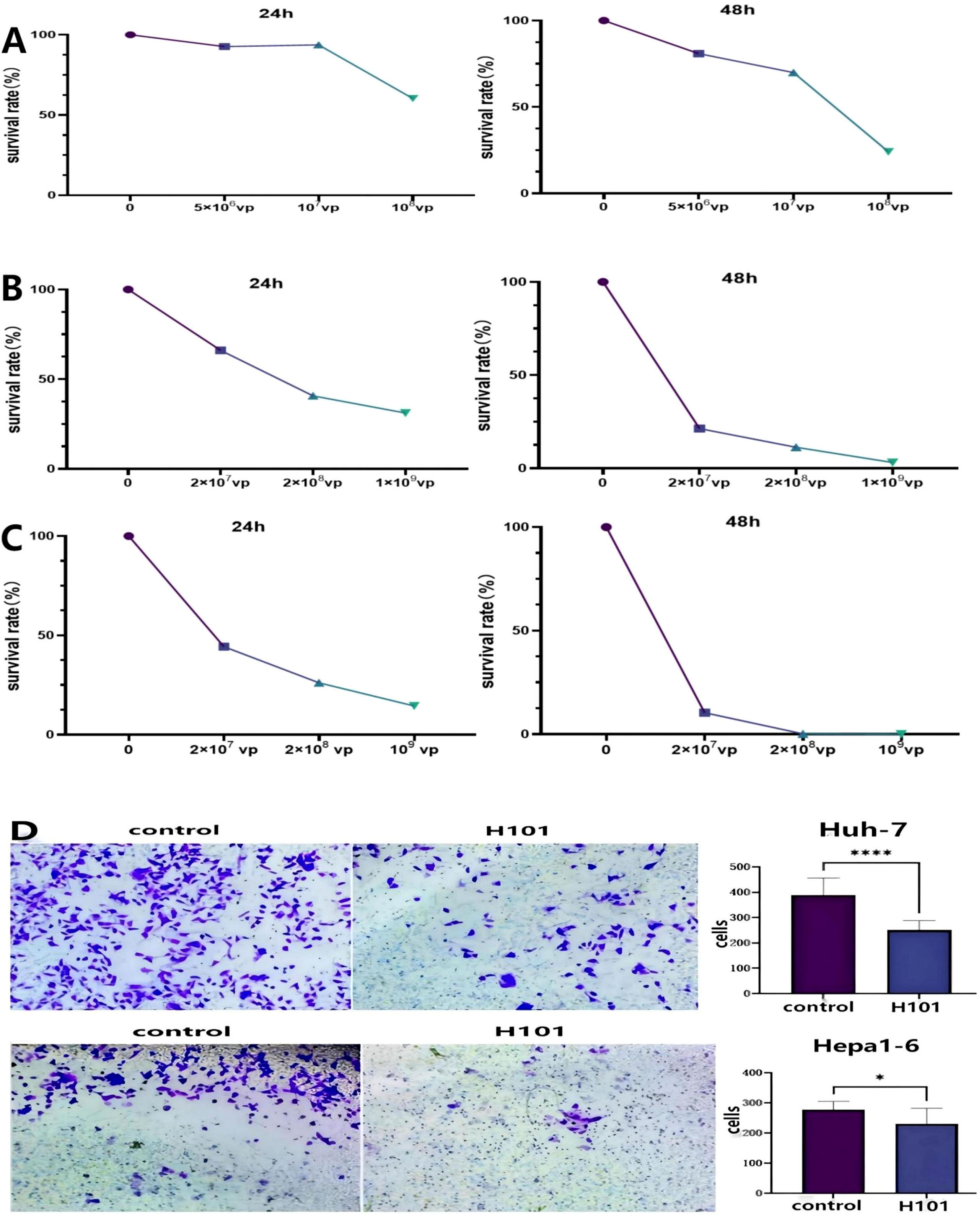
H101 inhibits the proliferation and invasion potential of HCC cell lines. **(A-C)** Cell viability was measured using the MTT assay in Hepa1-6 **(A)**, Huh-7 **(B)** and H22 **(C)** cells. **(D)** Transwell assay was performed to detect the effect of H101 on the invasive potential of the Huh-7 and Hepa1–6 cells. Transwell chambers coated with Matrigel. The upper chamber was loaded with 200 μL of cell suspension (H101 concentration at 4×10^9^/mL in the treatment group), while the lower chamber was filled with 600 μL of complete medium supplemented with 10% FBS. The statistical analysis was performed by unpaired t test. Data are presented as mean values ± SD. *p <0.05; ****p <0.0001.

To investigate the impact of H101 on HCC, H101 was co-incubated with Hepa1-6, Huh-7, and H22 cell lines, and cell viability was assessed by MTT assay at 24 and 48 h post-incubation. The results demonstrated that H101 exerted concentration- and time-dependent inhibitory effects on the viability of Hepa1-6, Huh-7, and H22 cells, with varying degrees of sensitivity observed among the three cell lines (**Figures 1A–C**). To investigate the impact of H101 on the invasion capabilities of HCC cells, *in vitro* transwell assays were performed following H101 co-incubation with Huh-7 and Hepa1–6 cells. The experimental results revealed that H101 significantly suppressed the invasive potential of both Huh-7 (p <0.0001) and Hepa1–6 cells (p = 0.0302) (**Figure 1D**).

### 3.2 H101-APA combination therapy exhibits enhanced anti-HCC effects

**Figure 2:**
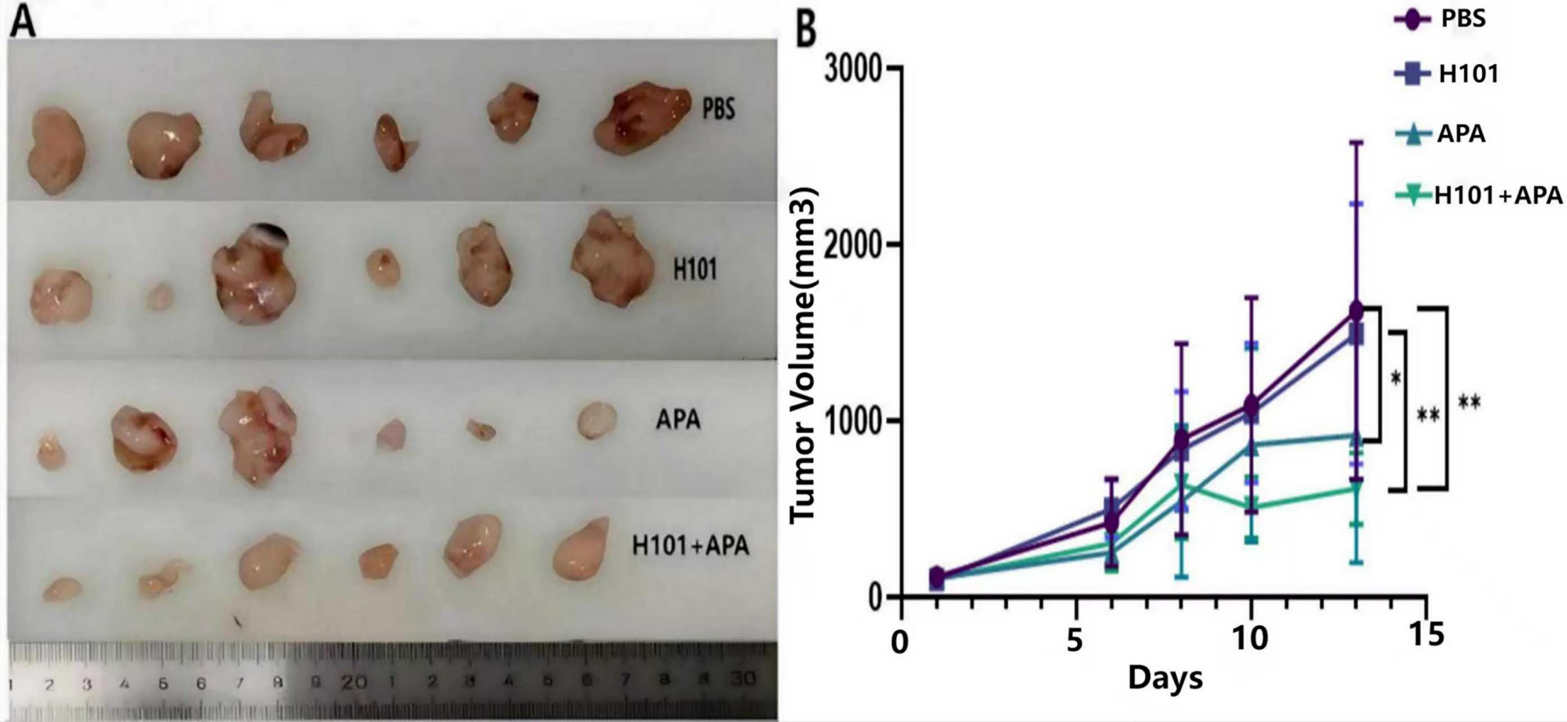
The tumor volumes were measured on D1, D6, D8, D10, and D13. Six C57BL/6N mice per group, with the PBS group serving as the control. **(A)** Tumor morphology across the four treatment groups; **(B)** Comparative curves of tumor volume in the four treatment groups. *p <0.05; **p <0.01.

To evaluate the differential therapeutic effects of H101 monotherapy, APA monotherapy, and their combination on HCC, we established HCC models using C57BL/6N mice. The APA monotherapy group was administered 100 μg APA via intraperitoneal (i.p.) injection. The H101 monotherapy group received 1×10^10^ viral particles (vp) of H101, administered via intratumoral injection at three distinct sites on D1 and D7. The combination therapy group received both treatments: 100 μg APA (i.p.) on D1, D4, D7, and D10, plus 1×10^10^ vp H101 (intratumoral injection) on D1 and D7. All the mice showed no obvious adverse reactions throughout the experimental period. On day 13, the mean tumor volume in both the APA monotherapy group and combination therapy group was significantly smaller than those in the PBS group (p <0.01) (**Figure 2**). In the combination group, the average tumor volume was slightly larger than the APA group from day 6 to day 8, and then became smaller than the APA group from day 10 to day 13. These results demonstrate that the combination therapy of H101 and APA achieves superior therapeutic efficacy in treating HCC.

### 3.3 H101 combined with APA elevated IFN-γ levels and CD8+ T cell infiltration in the TME

**Figure 3:**
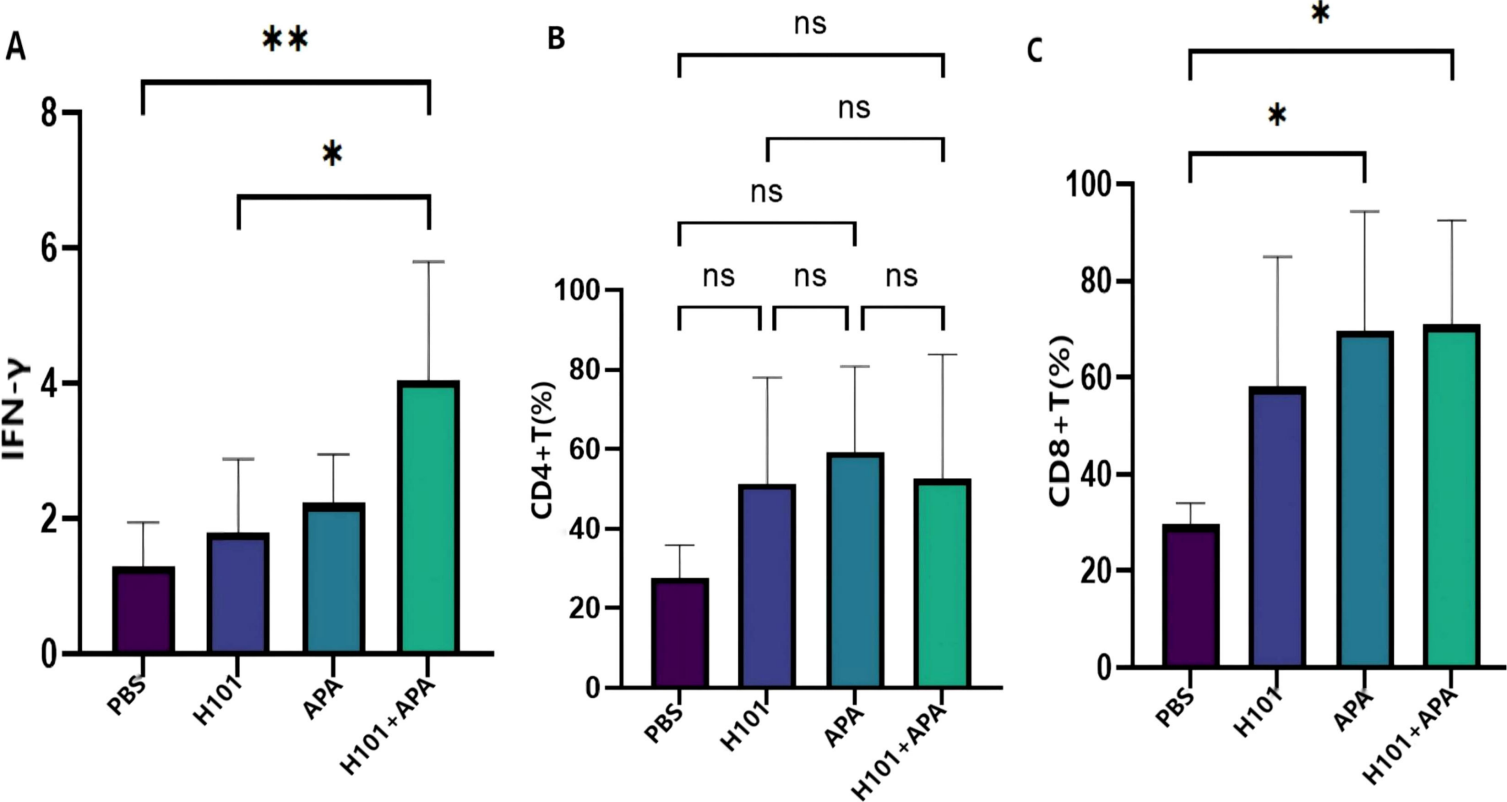
The effect of four treatment groups on IFN-γ levels, CD4+ T cell proportion and CD8+ T cell proportion. **(A)** The level of IFN-γ was detected by ELISA. **(B, C)** The proportion of CD4+ and CD8+ T cells were detected by flow cytometry. The statistical analysis was performed by one-way ANOVA with Tukey’s correction. Data are presented as mean values ± SD. *p <0.05; **p <0.01; ns, not significant.

To investigate the differential impact of different treatment groups on the TME, we detected IFN-γ levels using ELISA and quantified CD4+ and CD8+ T cell populations through flow cytometry. Results demonstrated that IFN-γ levels in the combination therapy groups were significantly elevated compared with control group (p = 0.0084) and the H101 group (p = 0.0252) (**Figure 3A**). Compared to the control group, the number of CD4+ and CD8+ T cells in the TME were increased to varying degrees in all treatment groups (**Figure 3B**), with significant increase of CD8+ T cells observed in both the combination therapy group (p = 0.0267) and the APA group (p = 0.0323).

### 3.4 IFN-γ exhibits cytotoxic effects on HCC cell and upregulates the gene expression of STAT1 and CD274 (PD-L1)

**Figure 4:**
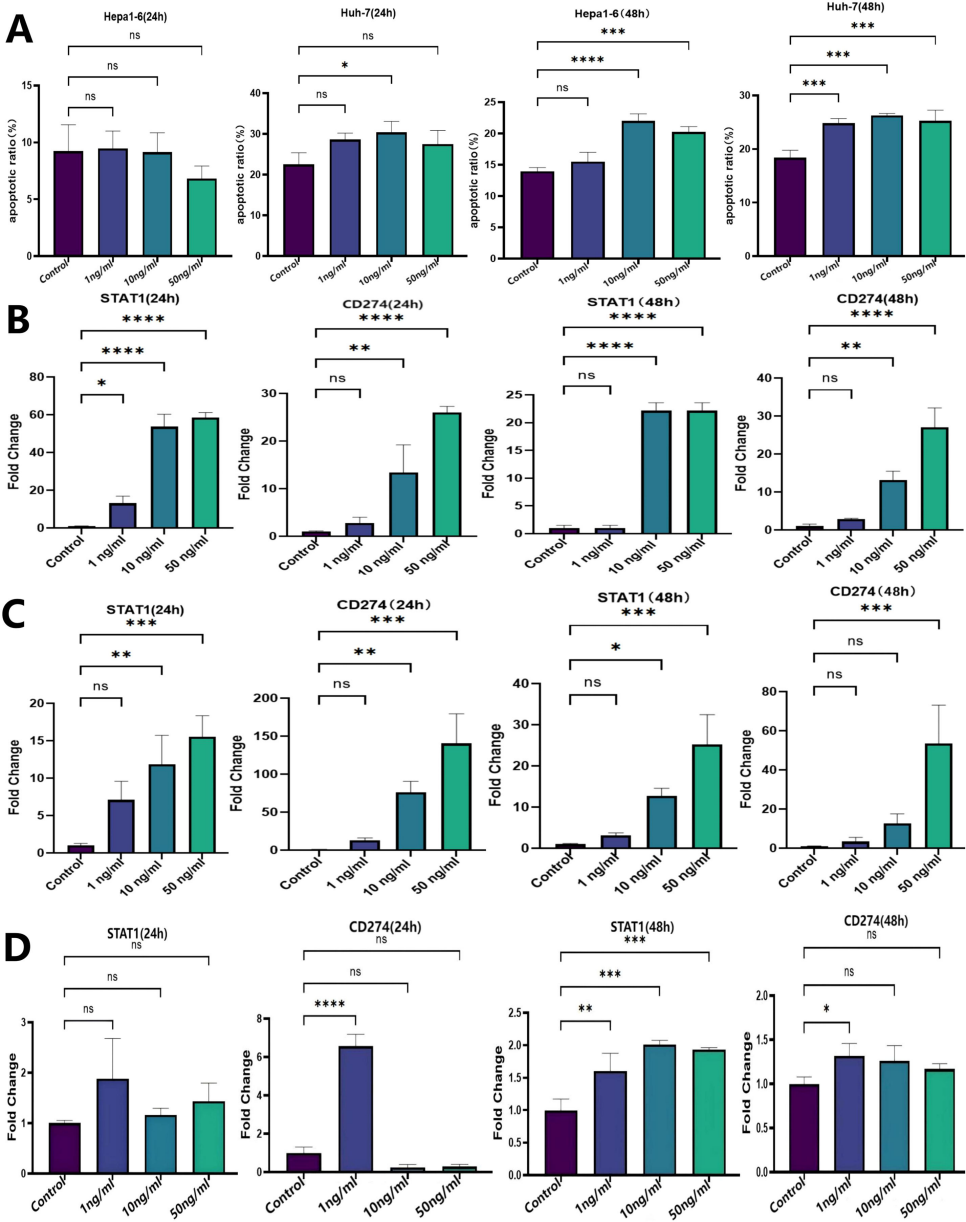
IFN-γ promotes apoptosis and enhances the gene expression of STAT1 and CD274 (PD-L1) in HCC cells. **(A)** Flow cytometry was performed to determine the cellular apoptotic rate. **(B-D)** The mRNA expression levels of STAT1 and CD274 (PD-L1) were measured by qPCR. IFN-γ was incubated with Hepa1-6 **(B)**, Huh-7 **(C)** and H22 **(D)** cells at three different concentrations (1 ng/ml, 10 ng/ml, 50 ng/ml). The statistical analysis was performed by one-way ANOVA with Tukey’s correction. Data are presented as mean values ± SD. *p <0.05; **p <0.01; ***p <0.001; ****p <0.0001; ns, not significant.

To explore the biological function of IFN-γ in HCC progression, Hepa1–6 and Huh-7 cells were incubated with different concentrations of IFN-γ, and the apoptotic rate was determined by flow cytometry. The results showed that IFN-γ promoted cell apoptosis of both Hepa1–6 and Huh-7 cells at concentrations of 10 ng/ml and 50 ng/ml following 48 h of incubation (**Figure 4A**). Additionally, Hepa1-6, Huh-7, and H22 cells were treated with varying doses of IFN-γ to detect the mRNA expression levels of its downstream molecules STAT1 and CD274 (PD-L1) by qPCR. The results demonstrated that IFN-γ significantly upregulated STAT1 and CD274 expression at both 24 and 48 h post-treatment (**Figures 4B–D**), indicating that IFN-γ induces the activation of the STAT1/PD-L1 signaling pathway in HCC cells.

### 3.5 Transfection with si-STAT1 reduces STAT1 and CD274 (PD-L1) expression at both the gene and protein levels

**Figure 5:**
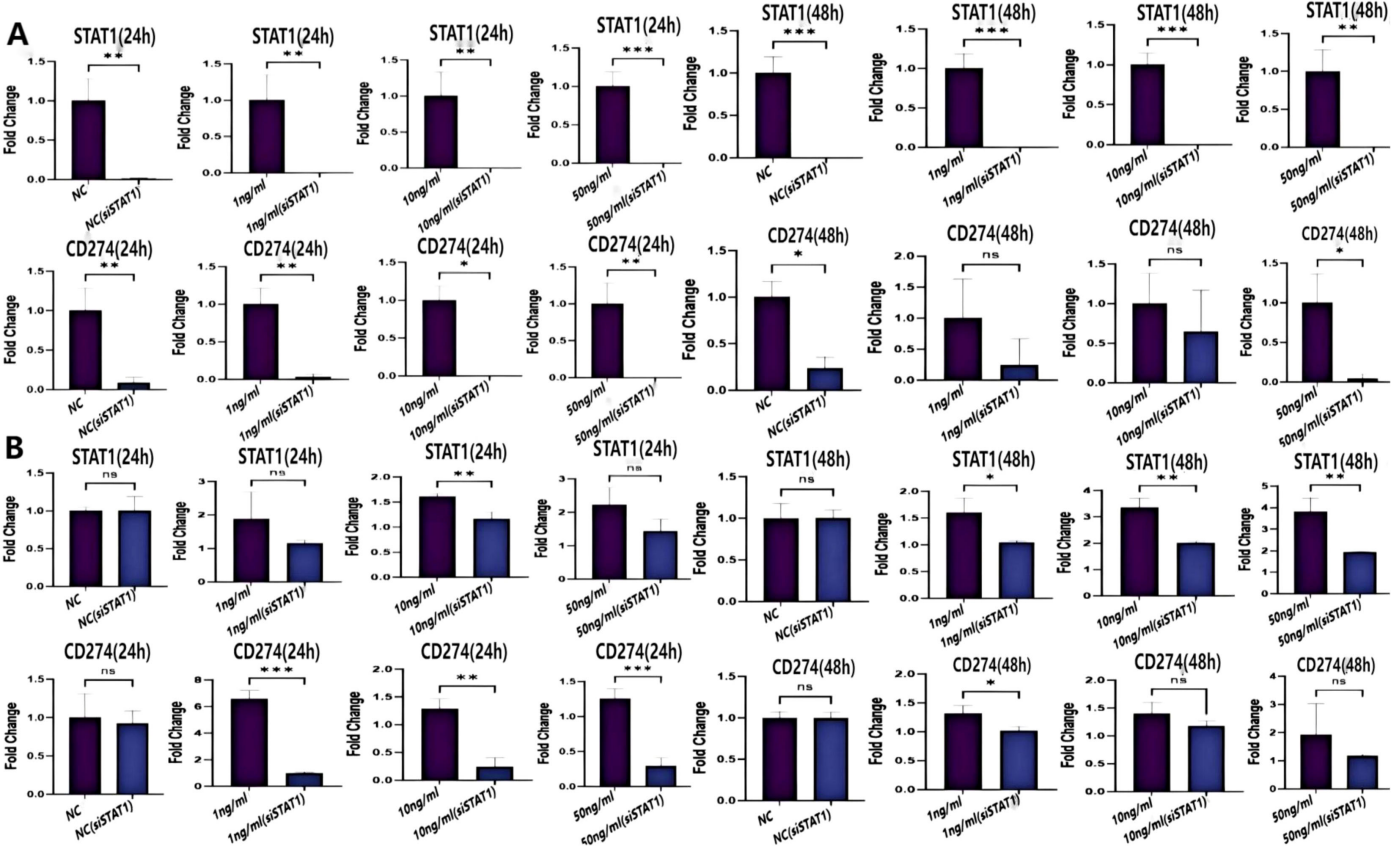
Transfection of si-STAT1 suppressed STAT1 and CD274 expression at the mRNA level in Huh-7 **(A)** and H22 cells **(B)**. The statistical analysis was performed by nonparametric T test. Data are presented as mean values ± SD. *p <0.05; **p <0.01; ***p <0.001; ns, not significant.

**Figure 6:**
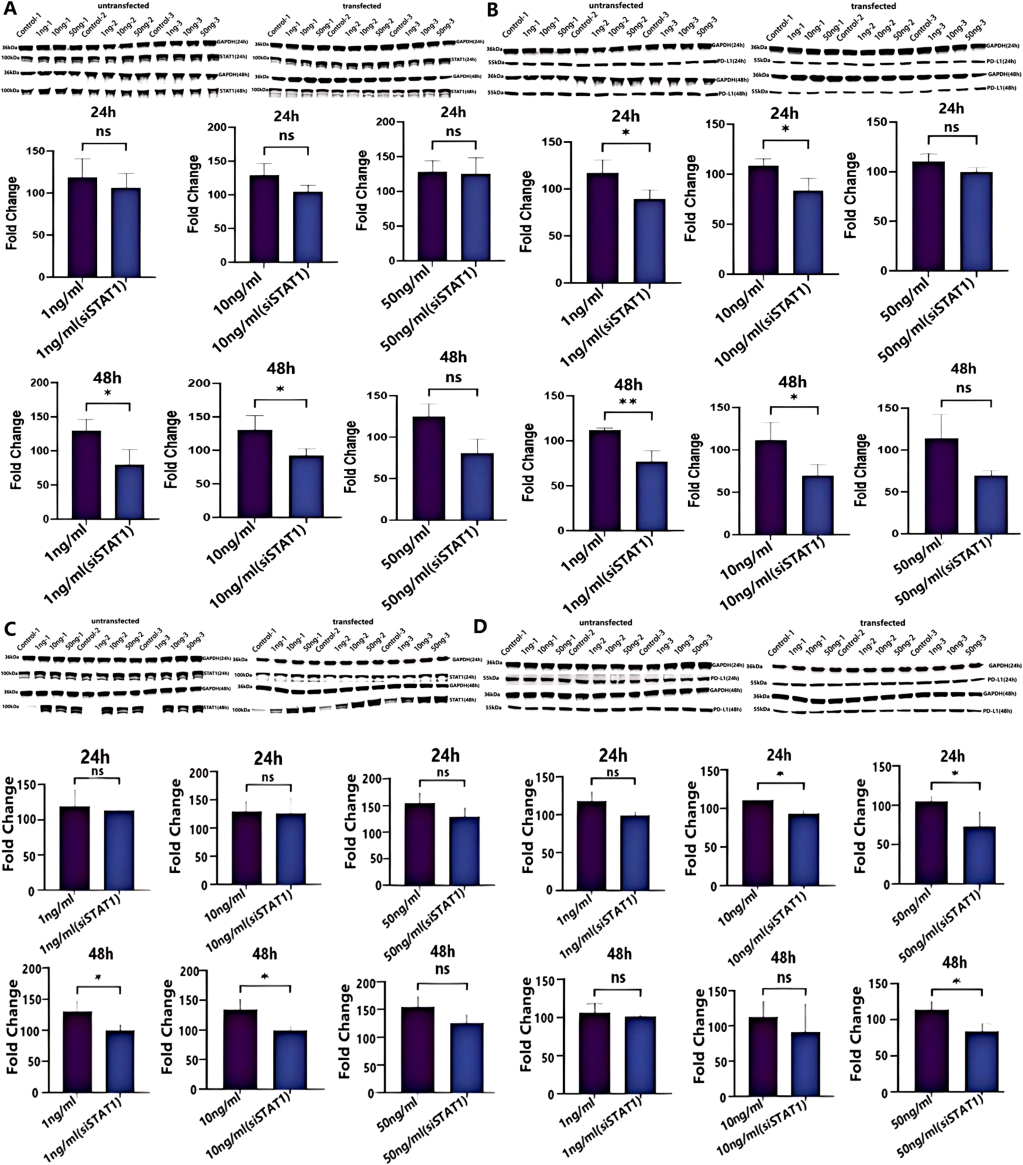
Transfection of si-STAT1 down-regulated the expression of p-STAT1 and PD-L1 proteins. **(A, B)** Western blot images showing the expression of p-STAT1 (100 kDa) and PD-L1 (55 kDa) proteins in Huh-7 cells before and after si-STAT1 transfection. **(C, D)** Western blot images showing the expression of p-STAT1 (100 kDa) and PD-L1 (55 kDa) proteins in H22 cells before and after si-STAT1 transfection. The molecular weight of the internal reference protein GAPDH is 36 kDa. The doses of IFN-γ used in all experiments were 1 ng/ml, 10 ng/ml, and 50 ng/ml. The statistical analysis was performed by nonparametric T test. Data are presented as mean values ± SD. *p <0.05; **p <0.01; ns, not significant.

To further validate the regulatory mechanism of the IFN-γ/STAT1/PD-L1 signaling pathway in HCC cells, si-STAT1 transfection was performed in Huh-7 and H22 cells. Compared to untransfected controls, STAT1 knockdown led to a marked reduction in the mRNA expression levels of both STAT1 and CD274 (**Figure 5**), confirming that si-STAT1 effectively suppresses CD274 expression. Subsequently, Western blot analysis was employed to further detect the expression changes of p-STAT1 and PD-L1 proteins in Huh-7 and H22 cells before and after si-STAT1 transfection. The results showed that the expression of p-STAT1 was significantly downregulated in si-STAT1-transfected Huh-7 cells after 48 h of treatment with 1 ng/ml (p = 0.0341) and 10 ng/ml (p = 0.0490) IFN-γ (**Figure 6A**). Meanwhile, PD-L1 expression was significantly downregulated after 24 h and 48 h of treatment with 1 ng/ml (p = 0.0408, p = 0.0350) and 10 ng/ml (p = 0.0072, p = 0.0455) IFN-γ (**Figure 6B**). In si-STAT1-transfected H22 cells, p-STAT1 expression was significantly downregulated after 48 h of treatment with 1 ng/ml (p = 0.0483) and 10 ng/ml (p = 0.0317) IFN-γ (**Figure 6C**). PD-L1 expression was significantly downregulated after 24 h of treatment with 10 ng/ml (p=0.0473) and 50 ng/ml (p = 0.0464) IFN-γ, as well as after 48 h of treatment with 50 ng/ml IFN-γ (p = 0.0353) (**Figure 6D**). These results indicated that under the same dose of IFN-γ treatment, compared with the untransfected control group, si-STAT1 transfection led to varying degrees of downregulation of p-STAT1 and CD274 (PD-L1) expression in both Huh-7 and H22 cells.

### 3.6 Transfection with si-STAT1 attenuates the anti-tumor efficacy of both APA monotherapy and the combination therapy of H101 and APA

**Figure 7:**
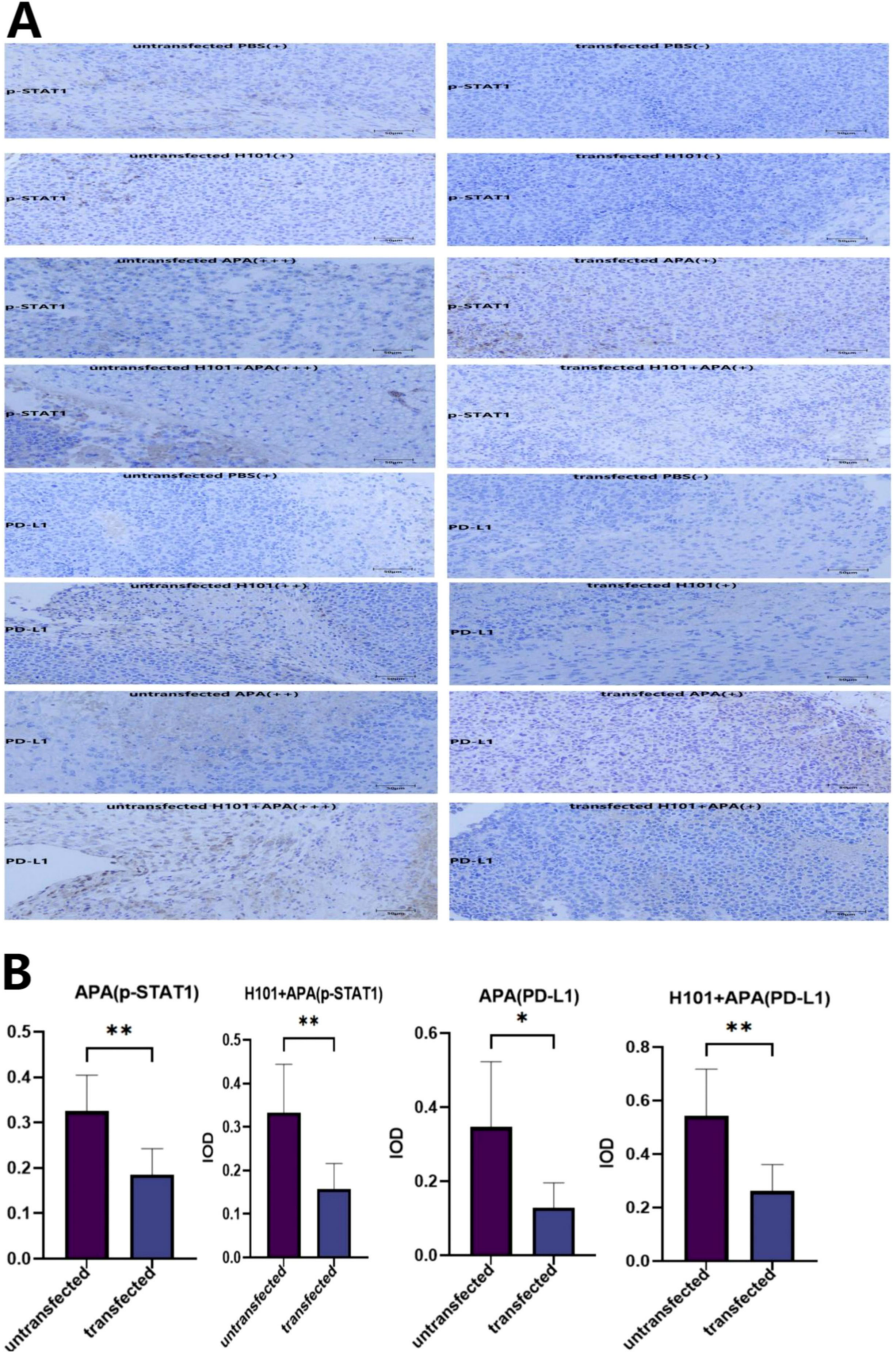
The protein expression levels of p-STAT1 and PD-L1 were detected by immunohistochemical staining. **(A)** Representative images of immunohistochemical staining (scale bar, 50 μm). The brown-stained cells are positive cells. **(B)** We used the IOD value calculation method for quantitative analysis of immunohistochemical differences (IOD, integrated optical density; IOD = stained positive area × average optical density). The statistical analysis was performed by nonparametric T test. Data are presented as mean values ± SD. *p <0.05; **p <0.01.

**Figure 8:**
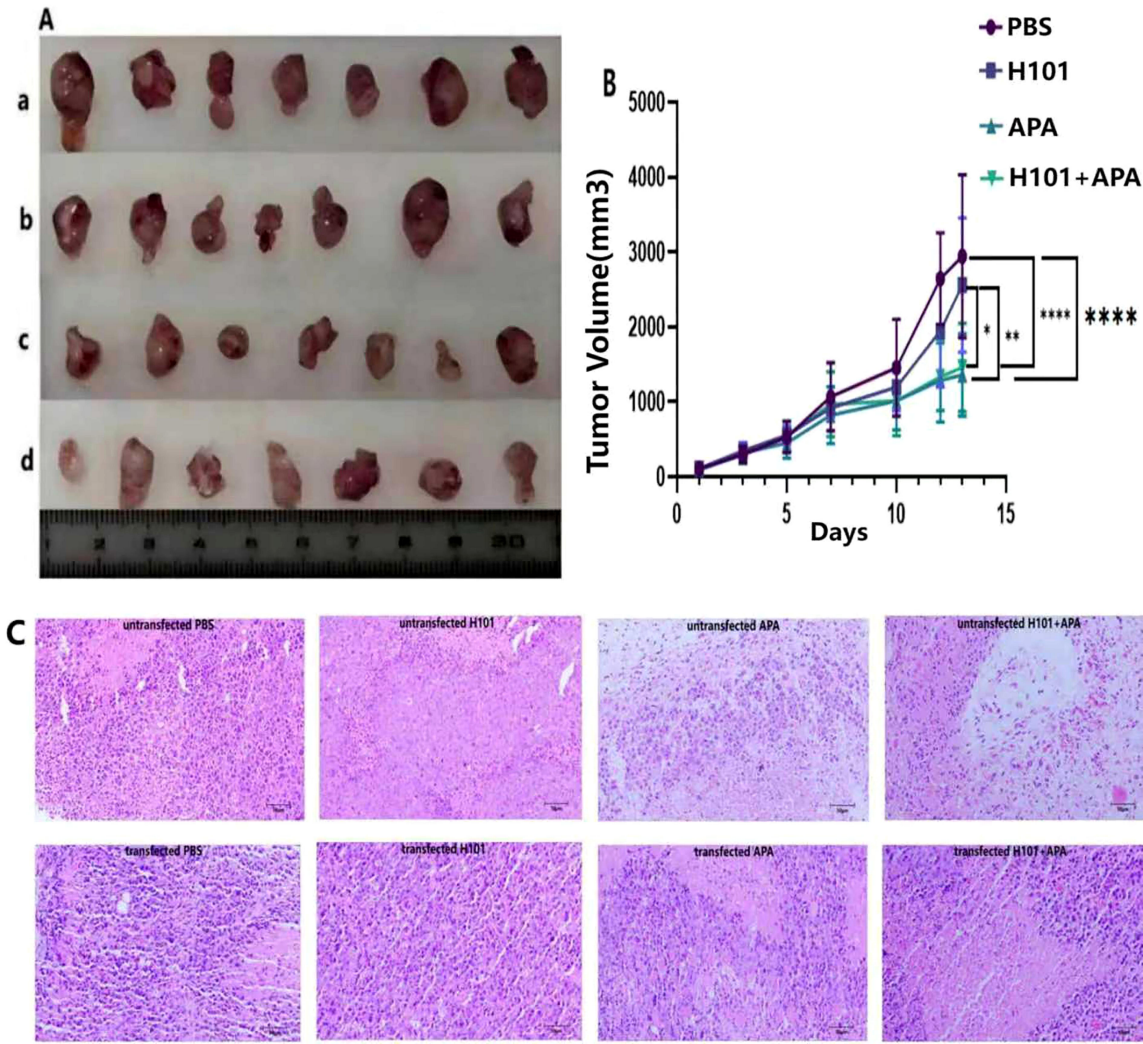
Transfection with si-STAT1 attenuated the anti-tumor efficacy. **(A)** Tumor morphology of si-STAT1-transfected C57BL/6N mouse HCC models following four treatments: PBS control **(a)**, H101 **(b)**, APA **(c)**, and H101+APA combination therapy **(d)**. Seven mice per group. Scale bars: 1 cm. **(B)** Comparison curves of tumor volume for the four treatment groups of si-STAT1-transfected C57BL/6N mouse models. **(C)** HE staining of the specimens isolated from experimental mice. Tumor cell density was semi-quantitatively scored based on the percentage of viable tumor cells in the tumor area: 0 (none), 1 (<25%), 2 (25%-50%), 3 (51%-75%), and 4 (>75%). The median scores of the surviving tumor cell densities in the PBS, H101, APA, and H101+APA groups before si-STAT1 transfection were 3, 2.5, 2, and 1 respectively. After transfection, they were 3.5, 3.5, 3, and 2 respectively. *p <0.05; **p <0.01; ****p <0.0001.

To further confirm the role of STAT1 in mediating the anti-tumor efficacy of H101-APA combination therapy, we established si-STAT1 transfected C57BL/6N mouse HCC models. Immunohistochemical analysis revealed that transfection with si-STAT1 downregulated the protein expression of both p-STAT1 and PD-L1 in tumor tissues compared to pre-transfection levels, and the differences were statistically significant in the APA group and the H101+APA group (p = 0.0058, p = 0.0064, p = 0.0170, p = 0.0066), further confirming the existence of STAT1/PD-L1 axis *in vivo* (**Figure 7**). APA monotherapy group and H101+APA combination therapy group still exhibited the strongest anti-tumor efficacy (p <0.0001) (**Figures 8A, B**). The HE staining results revealed differences in tumor cell density among different treatment groups (**Figure 8C**). Regardless of si-STAT1 transfection status, the combination therapy group showed the lowest tumor cell density, accompanied by higher tumor cell necrosis. Compared to the untransfected group, the anti-tumor efficacy of H101 monotherapy, APA monotherapy, and H101+APA combination treatments was all attenuated, as evidenced by increased tumor cell density in all treatment groups. These findings are consistent with the observed therapeutic effect, indicating a significant negative correlation between the therapeutic effect and the tumor cell density: the better the therapeutic effect, the fewer viable tumor cells observed in the tissue sections.

### 3.7 Transfection with si-STAT1 modulates the TME *in vivo* during H101-APA combination therapy

**Figure 9:**
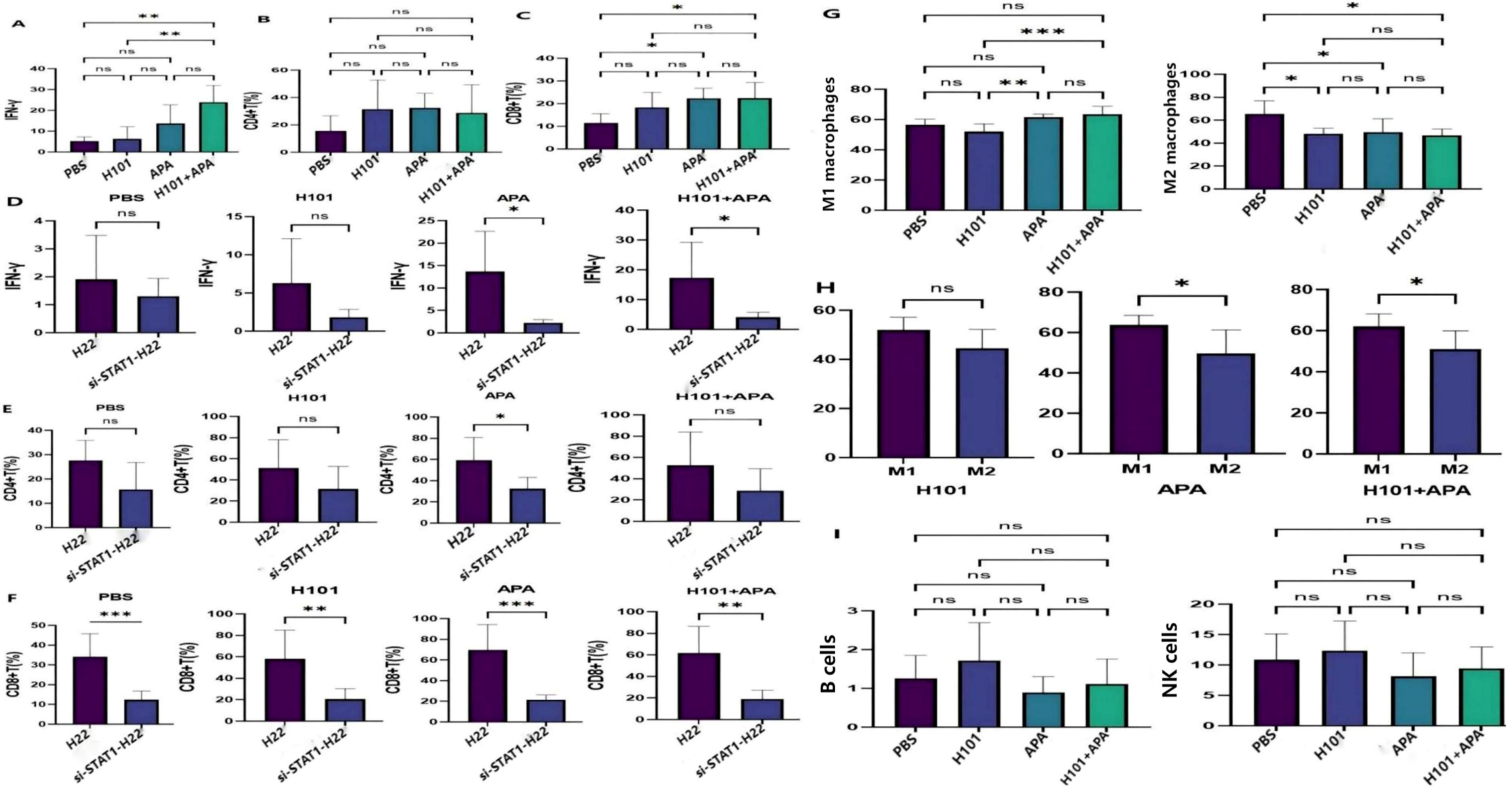
The influences of si-STAT1 transfection on the TME. **(A)** IFN-γ level was detected by ELISA. The statistical analysis was performed by one-way ANOVA with Tukey’s correction. **(B, C)** CD4+ T and CD8+ T cell infiltration levels were detected by flow cytometry. Statistical comparisons among multiple groups were performed using one-way ANOVA with Tukey’s correction, while the unpaired t-tests were employed for comparisons between two groups. **(D)** The level of IFN-γ was detected by ELISA before and after si-STAT1 transfection. **(E, F)** The infiltration levels of CD4+ T and CD8+ T cells were detected by flow cytometry before and after si-STAT1 transfection. **(G-I)** Flow cytometry plots of CD86+ M1, CD206+ M2 macrophages, NK cells, and B cells. Statistical comparisons among multiple groups were performed using one-way ANOVA with Tukey’s correction, while the unpaired t-tests were employed for comparisons between two groups. Data are presented as mean values ± SD.*p <0.05; **p <0.01; ***p <0.001; ns, not significant.

To further elucidate the underlying mechanism by which si-STAT1 attenuates the anti-tumor efficacy of H101-APA combination therapy, we explored the impact of si-STAT1 transfection on the TME *in vivo*. Results demonstrated that IFN-γ levels were significantly elevated in the combination therapy group compared with both the H101 group (p = 0.0012) and PBS group (p = 0.0016) (**Figure 9A**). The infiltration levels of both CD4+ T and CD8+ T cells were increased, with only CD8+ T cells showing statistically significant differences in PBS vs. H101 groups (p = 0.0327) and PBS vs. combination therapy groups (p = 0.0428), respectively (**Figures 9B, C**). Compared to untransfected controls, si-STAT1-transfected groups exhibited significantly reduced IFN-γ levels in the APA and combination therapy groups (**Figure 9D**). Compared to untransfected controls, si-STAT1-transfection led to reduced infiltration levels of both CD4+ T and CD8+ T cells. The most pronounced reduction in CD4+ T cell infiltration was observed in the APA group (p = 0.0138), while CD8+ T cell infiltration was significantly decreased across all treatment groups (PBS: p = 0.0138; H101: p = 0.0054; APA: p = 0.0003; H101+APA: p = 0.0013) (**Figures 9E, F**). Notably, the infiltration levels of CD86+ M1 macrophages were significantly elevated in H101 vs. APA groups (p = 0.0037) and APA vs. combination groups (p = 0.0012), respectively (**Figure 8G**). Conversely, the infiltration levels of CD206+ M2 macrophages were markedly decreased in H101, APA, and combination therapy groups compared with the PBS control group (p = 0.0297, p = 0.0358, and p = 0.0171) (**Figure 9G**). Compared to M1 macrophages, the levels of M2 macrophages were significantly decreased in both APA and combination therapy groups (**Figure 9H**). No significant differences in B cells and NK cell levels were observed among four groups (**Figure 9I**). Collectively, these results indicate that the H101-APA combined therapy may reshape the TEM partially through the IFN-γ/STAT1/PD-L1 signaling pathway, and si-STAT1 transfection could abrogate this modulatory effect, thereby weakening anti-tumor efficacy.

## 4 Discussion

In recent years, ICIs-based therapies have become a critical component in the treatment of advanced HCC (18). However, the presence of an immunosuppressive TME in HCC results in a response rate of less than 20% for ICIs monotherapy in HCC patients (19). Combination therapy represents a novel treatment strategy for patients developing resistance to conventional treatment modalities (20). OVs preferentially infect and destroy cancer cells, thereby inducing immunogenic cell death, increasing immune cell infiltration and overcoming immunosuppression within the TME (21). Combining ICIs with OVs has been considered the most promising opportunity in HCC (22).

In this study, H101 monotherapy demonstrated moderate antitumor efficacy, followed by APA monotherapy, and the combination of H101 and APA exhibited the best therapeutic efficacy in the HCC mouse model. The combination therapy group elevated the levels of IFN-γ, STAT1, CD274, CD4+ T cells (with no statistical significance), and CD8+ T cells in the TME. Transfection with si-STAT1 significantly decreased the protein expression of p-STAT1 and PD-L1, attenuated the anti-tumor efficacy, and reshaped the TME *in vivo*.

CD8+ T cells represent the primary effector cells in anti-tumor immune responses. They can collaborate with T helper 1 (Th1) cells to release perforin, IFN-γ, granzymes, and tumor necrosis factor (TNF)-α, thereby mediating potent anti-tumor immunity (23). Accordingly, T cell exhaustion has emerged as a key factor that limits the duration of clinical response to ICI therapy (24, 25). Recent studies have found thatCD4+ T cells play a critical role in the development and maintenance of effective anti-tumor immunity, even in immunotherapies specifically designed to activate CD8+ T cell (26). Bos et al., have shown that IL-2 produced by tumor-specific CD4+ T cells promotes the proliferation of CD8+ T cells and upregulates granzyme B expression (27). Additionally, IFN-γ-induced chemokines secreted by CD4+ T cells enhance the recruitment of CD8+ T cells into tumors. In certain contexts, CD4+ T cells inhibit tumor growth not only by aiding CD8+ T cells differentiation but also through direct cytolytic effector functions. Upon encountering antigen stimulation, CD4+ T cells secrete IFN-γ, which exhibits anti-tumor activity. Within the TME, IFN-γ upregulates the expression of both MHC class I and MHC class II molecules, thereby enhancing antigen presentation by antigen-presenting cells (APCs) (28). CD4+ T cells can also differentiate into cytotoxic CD4+ CTLs that directly mediate anti-tumor effects (29, 30). IFN-γ is primarily secreted by T lymphocytes, natural killer (NK) cells, and APCs, (including monocytes, macrophages, and dendritic cells). As a central regulator of the immune microenvironment, IFN-γ enhances host defense through mechanisms such as activating macrophage functions, upregulating MHC molecule expression, and driving Th1 differentiation. A transcriptomic study identified IFN-γ derived from CD8+ T cells as a master regulator of the TME, with its extensive regulatory influence earning it the designation of a “global orchestrator” of the immune microenvironment (31). IFN-γ can directly act on tumor cells to induce cellular senescence (32). Additionally, it targets tumor stroma and endothelial cells to promote anti-tumor immune responses (33). The efficacy of ICIs is at least partially attributable to IFN-γ signaling and tumors unresponsive to therapy frequently harbor mutations in the IFN signaling pathway (34–36). Our study indicated that the combination of H101 and APA enhanced the infiltration of CD4+ T cells in the TME, as well as the expression of IFN-γ. These findings suggests that H101 remodels the immunosuppressive TME, thereby potentiating the antitumor efficacy of APA. Furthermore, these results confirm the critical role of IFN-γ in immune regulation.

Various pro-inflammatory cytokines produced by tumor-infiltrating immune cells contribute to the establishment of an immunosuppressive TME, in part by upregulating PD-L1 expression and thereby promoting tumor progression. IFN-γ act as the primary inducer of PD-L1 expression via the JAK–STAT signaling axis and may influence cancer immune surveillance and the responsiveness to anti-PD-1/PD-L1 immunotheray (35, 37). Recent studies have further revealed that the duration of IFN-γ signaling is critical: prolonged IFN-γ exposure may elicit pro-tumorigenic immune programs or drive tumor hyperprogression (38–40). Patients with high PD-L1 expression (>1%) demonstrate longer survival than those with PD-L1 expression <1%, supporting PD-L1 as a promising predictive biomarker for anti-PD-1/PD-L1 therapies (41–43). Collectively, IFN-γ exhibits a dual role in tumor immunology: it can induce immunogenic cell death in tumor cells, while simultaneously promoting PD-L1 expression. Overexpressed PD-L1 binds to the PD-1 receptor on T cells, inhibits T-cell effector function, and establishes an immunosuppressive microenvironment, that facilitates tumor immune escape (44, 45). In the present study, H101+APA combination therapy upregulated IFN-γ expression, and IFN-γ enhanced the expression of both STAT1 and PD-L1 in HCC cells. In HCC mouse models with si-STAT1 transfection, the antitumor efficacy of H101 monotherapy, APA monotherapy, and H101+APA combination therapy was significantly attenuated. STAT1 knockdown downregulated PD-L1 expression, consequently impairing therapeutic effect of APA. Furthermore, si-STAT1 transfection reduced CD4+ and CD8+ T cell infiltration in the TME along with decreased IFN-γ production compared to non-transfected controls.

The balance between M1- and M2-polarized tumor-associated macrophages (TAMs) in the TME plays a pivotal role in tumorigenesis and progression. M1-TAMs exert antitumor effects, whereas M2-TAMs typically promote tumor growth (46, 47). Xiao et al., demonstrated significant infiltration of CD86+ M1-TAMs in HCC tissues, and high CD86+ M1-TAM density was correlated with suppressed tumor progression, prolonged overall survival, and improved disease-free survival (48). Moreover, M1-TAM density showed positive correlation with CD8+ T cell infiltration. IFN-γ also serves as a crucial regulator that drives macrophage polarization toward the M1 phenotype. In this study, we quantified the proportions of M1 macrophages, M2 macrophages, B cells, and NK cells in the TME. The H101+APA combination group and APA monotherapy group exhibited significantly higher M1 macrophage infiltration compared to H101 monotherapy, and markedly decreased M2 macrophage infiltration compared to PBS group, while no significant differences were observed in B cells or NK cells. The enhanced IFN-γ expression induced by H101+APA combination may represent a key mechanism underlying the increased M1 macrophage polarization observed in our model.

Several limitations of the present study should be acknowledged. First, this study was performed as a proof-of-concept investigation guided by the 3R principles (Replacement, Reduction, Refinement) for animal welfare and ethical requirements, which resulted in a relatively small sample size, a short observation period, and the absence of long-term survival analyses. Although these settings allowed us to explore the therapeutic efficacy and underlying mechanism while minimizing animal usage and distress, they restricted the translational strength of the present findings. Second, we employed a subcutaneous HCC model, which was convenient for standardized evaluation and mechanistic exploration but cannot fully recapitulate the native tumor microenvironment and clinical progression of HCC. Third, immune characterization was largely restricted to quantitative and phenotypic profiling of immune cell infiltration, lacking in-depth functional assessments including T-cell cytotoxicity, T-cell exhaustion markers, and macrophage functional states beyond surface phenotypic markers. Fourth, although STAT1 silencing supported the involvement of the IFN−γ/STAT1 pathway, definitive causal evidence was not established due to the absence of stable gene knockout or cytokine blockade experiments. Finally, this study relied exclusively on H101 without comparative analyses of other viral platforms. Given the substantial differences in tropism, immunogenicity, and replication dynamics among distinct oncolytic viruses, it remains unclear whether the identified IFN−γ/STAT1/PD−L1 signaling axis is H101−specific or represents a generalizable mechanism of oncolytic virotherapy. These limitations should be considered when interpreting the mechanistic conclusions and translational potential of this study. Future investigations will adopt orthotopic tumor models, perform long-term survival studies with appropriate sample sizes, and integrate comprehensive immune functional assays to further validate and extend our findings. Comparative studies using multiple oncolytic viral platforms will be performed to clarify whether the IFN−γ/STAT1/PD−L1 signaling axis represents a vector-specific mechanism or a conserved regulatory principle of oncolytic virotherapy in the future.

In summary, this study demonstrates that the combination of H101 and APA enhances anti-HCC efficacy, primarily by increasing the infiltration of CD8+ T cells and CD4+ T cells in the TME, boosting IFN-γ expression, and promoting M1 macrophage polarization, which is partially regulated by the IFN-γ/STAT1/PD-L1 signaling pathway. Given the current clinical limitations of ICIs in HCC treatment, the strategy of combining H101 with APA represents an exciting novel approach in cancer immunotherapy, with promising potential for future clinical applications.

## Data Availability

The raw data supporting the conclusions of this article will be made available by the authors, without undue reservation.
